# Indexing Quit-Smoking Interest among Norwegian Smokers 2019-2021

**DOI:** 10.1155/2023/9536270

**Published:** 2023-02-13

**Authors:** Gunnar Sæbø, Karl Erik Lund

**Affiliations:** Department of Alcohol, Tobacco and Drugs, Norwegian Institute of Public Health, PO Box 222, 0213 Skøyen, Oslo, Norway

## Abstract

Even if smoking prevalence is declining in several western countries, continued smoking cessation is required to reduce tobacco-related harms and to achieve future goals of smoke-free societies or the tobacco endgame. But how many of the current smokers want to quit? Estimates vary and depend on the type of question asked. We investigate how a pooled sample of Norwegian smokers (*N* = 1321) is distributed over four indicators of interest in quitting: (i) degree of desire to quit, (ii) prediction of future smoking status, (iii) reported plan for quitting smoking, and (iv) statements on previous attempts to quit. Based on these variables, we constructed an index. One-third of the smokers (32.6%) was categorized as having a high or very high interest in quitting. However, nearly half of the smokers (47.8%) had low or very low interest in quitting. Like several other countries, Norway has legislated a vision of a smoke-free society and, under the government's plans; this goal will be achieved by intensified use of structural measures such as tax hikes, tighter restrictions on outdoor smoking, and reduced availability of cigarettes. For the third who want to quit smoking, such constraints on their behaviour may help them to pursue their desire to quit. However, for the half who want to continue smoking, these measures may not be helpful but instead be experienced as a loss of welfare, less freedom to act, and increased social disqualification.

## 1. Introduction

Over recent decades, the proportion of smokers in most western countries has decreased sharply [1]. Smoking has gained negative symbolic meaning [2, 3], the user group has undergone a social declassification [4–6], and its behaviour is carried out in an increasingly tobacco-hostile social environment [7, 8]. Studies show that many smokers evaluate their own behaviour negatively [9] and feel stigmatized [10, 11]. Moreover, studies on risk perceptions show that smokers are well informed about the fact that continued smoking can cause deterioration to their own health [12, 13]. Among the remaining smokers in the population, external social pressure and internal health-related motivation may have induced a desire to quit, a self-prediction of a smoke-free future, concrete planning for a quit attempt or recent quit attempts.

On the other hand, smokers also report positive features that help maintain the behaviour. In the classic books, *Cigarettes Are Sublime* [14] and *Nicotine: A Love Story Up in Smoke*” [15], the authors describe favourable aspects of smoking that, for them, suppress interest in quitting. Here, smoking is about passion, balance, and harmony. Cigarettes also function as a social marker of distinction. The fact that you smoke, how you smoke and with whom you choose to smoke can be used to communicate identity, individuality and placing in the social landscape [16, 17]. Cigarettes may work as social crutches in unfamiliar surroundings, and smoking is a ritual act that can create a sense of community and bonding. As a psychoactive substance, nicotine can provide a sense of well-being and stimulate cognitive functions [18]. Cigarettes can also act as a reward, provide comfort, and relieve stress. Smoking can represent normative transgression and subcultural resistance and express style, sociality, joyful experiences, and cultural capital [19–21]. Some smokers may therefore have a weak desire to quit, much less have made concrete plans, or attempts to quit, and consider themselves smokers in the future, as of now.

In between the two segments of smokers that are ready to quit and those who definitely will not quit, we find the ambivalent smokers, who have an underlying desire to quit but at the same time wish to continue smoking [22–24]. They balance the negative consequences of smoking against the positive features without tipping in a particular direction, or their preferences in respect of quitting may be unstable [25].

There are varying estimates of the size of these three smoker segments in the literature. Quit-smoking interest can be extracted from various questions, which separately may produce very different results [26–29]. This complexity suggests the use of multiple indicators in an index that will produce a more robust and valid measure than the individual items ([30] : 123-136). As far as we know, no index measurement of interest in quitting smoking based on multiple indicators has been previously published.

In this article, we have examined how smokers are distributed on four indicators of interest in quitting: (i) expressed degree of desire to quit, (ii) prediction of future smoking status, (iii) reported plan for quitting smoking, and (iv) statements on previous quit attempts. Based on these items, we constructed an index for the degree of interest in quitting, compared scores for subgroups of smokers, and mapped how smokers were distributed on the index. As smokers in economic vulnerable situations tend to smoke more and quit less often than more privileged smokers, smoking cessation behaviours and outcomes may also be associated with sociodemographic status, which consequently needs to be controlled for.

## 2. Materials and Method

### 2.1. Materials

We use the data collected on behalf of the Norwegian Directorate of Health in connection with the Stoptober campaign [31]. This is an annual nationwide media campaign that motivates smokers and snus users to make an attempt at quitting during the month of October and stay tobacco-free for the following 28 days. Before and after the campaign, Mindshare Norway administer web surveys among tobacco users, which includes questions on intentions to quit and quit activity. We have analyzed data collected prior to the campaigns in 2019, 2020, and 2021.

Invitation for participation was sent by email to those who were preregistered as smokers or snus users in a national consumer web panel consisting of 85 000 people, administered by Norstat—a leading independent European data collector for market research. This sample is not based on self-recruitment. Approximately 80% of the panel participants were recruited through invitation by telephone, some were also recruited via other web surveys and Facebook.

The sample was randomly selected from the panel (4 559 panellists were contacted in 2019, 7 799 in 2020, and 11 075 in 2021) but was monitored in order to be able to compensate for any underrepresentation of different population segments based on combinations of age, gender, and region. When incoming responses reached a predesired number (500 tobacco users in 2019, 700 in 2020 and 2021), data collection was terminated (see table A in supplementary materials for annual gross respondence and distribution of respondents by smoking status).

The responses were given via a computer, smartphone, or tablet. Respondents were quarantined so as to exclude the same person from responding two years in a row. Participation in the survey provided the respondent with points that could be accumulated and, on reaching a certain number, be exchanged for a prize.

The inclusion criterion for our study was regular or irregular use (daily or occasional) of cigarettes, roll-your-own tobacco, cigars, cigarillos, or pipe tobacco. In the final pooled sample, there were 1377 smokers (Table 1). Our reporting is based on the 1321 smokers who responded to all four indicators of interest in quitting.

### 2.2. Measures

Smoking status was determined with the question “Do you smoke (Cigarettes, roll-your-own-tobacco, pipe, cigars/cigarillos)?” The response options were yes, daily, yes, occasionally, and no, I have quit. Respondents who had quit smoking since preregistration in Norstat but nevertheless answered the question on interest in quitting, were excluded from our analyses (see table A, supplementary materials).

The Norwegian Directorate of Health's questionnaire included questions with wordings often used in questionnaires that measure smokers' interest in quitting smoking, developed in the wake of the influential “stages of change” model [32]—for instance, the “Readiness and motivation to quit scale” [28], the “Reasons for quitting scale” [33], and the “Willingness to quit scale” [29]. These questions are also often used in reports from the US Department of Health and Human Services [34] and Centers for Disease Control and Prevention [27].

In our analysis, we have utilized four “stand-alone” indicators of smoking cessation from the Norwegian Directorate of Health's questionnaire. In a direct question, the smokers were asked to indicate their level of *desire to quit* (Table 2) on a scale from 1 (to a small degree) to 5 (to a very high degree). The wording of the question was “To what degree would you say that you want to quit smoking?” In previous studies, the desire to quit has proven to be a good predictor of future attempts to quit [30, 35] and is, for instance, included in the “Motivation to stop scale” [36].

The questionnaire also contained three indirect indicators of desire to quit. *Prediction of future smoking status* (Table 3) was measured by combining answers from two questions: initially, smokers were asked to answer the question “Do you see yourself as a non-smoker in the future?” with the options: yes, no, and do not know. Smokers who answered “yes” were asked the follow-up question “When do you see yourself as a non-smoker?” with the following response categories: within 12 months, 1-2 years, 3-4 years, in 5 years or later, and do not know. Prediction is a nonbinding statement about future behaviour and differs from an intention that contains a greater degree of planning and justification [37]. Nevertheless, studies have found that prediction regarding future smoking status is correlated with attempts to quit [26, 38, 39].

*Plans to quit* (Table 4) was measured by the question “Do you have plans to try to quit smoking?” The response options were as follows: I am considering quitting within the next month, I am considering quitting within 1-3 months, I am considering quitting within 4-6 months, I am considering quitting, but not within the next 6 months, I am not considering quitting smoking, and I do not know. Action planning has been associated with quitting smoking [40, 41] and of particular importance has been so-called implementation intentions [42, 43], where the smoker has related plans to quit and prepared for a critical situation of relapse.

Time since the last *attempt to quit* (Table 5) was assessed by combining responses to two questions. Initially, smokers were asked the question “Have you tried to quit smoking?” The answers were as follows: yes (graded by the number of times), no—I have never tried, and I do not remember. Respondents who answered “yes” were asked the follow-up question: “When did you last try to quit smoking?” with the options: last month, 1-3 months ago, 4-6 months ago, 7-12 months ago, 1-2 years ago, more than 2 years ago, and do not remember. Previous studies have shown that the time interval from the last attempt at quitting is associated with future attempts to quit smoking [44–46].

### 2.3. Construction of the Index

The response categories on the four above-mentioned cessation questions can be ranked according to the degree of interest in quitting smoking expressed by the respondents. In the last column of Tables 2–5, we have entered ranking scores that range from 0 (lowest level of desire to quit) to 5 (highest level of desire to quit). Our assignment of ranking scores was inspired by Crittenden et al. [28] and Pechacek et al. [47]. There are different ways of treating “do not know” answers. In some scales, respondents with such answers are excluded. We opted for a procedure whereby the numerical value for “do not know” answers in Tables 2–4 and “do not remember” answers in Table 5 were assigned a score that was higher than the extreme value for low interest in quitting.

An ordinal distribution of the categories of answers makes it possible to construct an additive index based on the four indicators for interest in quitting—one direct (Table 2) and three indirect (Tables 3–5). The value a respondent receives in the index is determined by the combination of ranking scores for the four indicators. The index value may thus vary between 0 (lowest level of desire to quit for all questions) and 20 (highest level of desire to quit for all questions). The index was given the same variation range as the four indicators (scale from 0 to 5) by dividing by four. Internal consistency was measured using Cronbach's alpha [48]. Differences in average index scores between subgroups of smokers were significantly tested with Independent Samples *T*-test. All analyses were carried out in SPSS version 27.

## 3. Results

### 3.1. Descriptive Characteristics of the Sample

Occasional smokers made up 44.8% of the population of current smokers. Other characteristics of the sample are shown in Table 1.

### 3.2. Desire to Quit

On the direct question concerning desire to quit smoking, respondents grouped into three segments. All together 38.2% of smokers stated that they wanted to quit smoking to a very great extent (16.0%) or a great extent (22.2%). About one in three smokers—32.6%—reported that they wished to quit smoking to a small extent (14.5%) or a very small extent (18.1%). The remaining 29.2% of smokers were in the middle of the scale (25.7%), or they did not know (3.5%). There was a significant difference in the response distribution between daily smokers and occasional smokers (Table 2).

### 3.3. Prediction of Future Smoking Status

Just over every fourth smoker (28.0%) assumed that they would become smoke-free within one year (Table 3). Almost the same number predicted that freedom from smoking would happen further into the future. In total, just over half of the smokers (54.3%) predicted a smoke-free future at one point in time or other—significantly more occasional smokers (62.6%) than daily smokers (48.4%). The remaining smokers did not imagine themselves to be nonsmoking in the future (17.2%), or they did not know (28.4%) what the future would bring.

### 3.4. Plans to Quit

Table 4 shows that 14.2% of the smokers were considering quitting smoking in the course of the next month and a further 15.0% within three months. Altogether, 27.2% were considering quitting smoking but only 4 or more months in the future. A quarter of smokers (25.3%) were not considering quitting smoking at all, while approximately one in five responded do not know/do not wish to answer. Many more daily smokers (63.9%) than occasional smokers (46.3%) were considering quitting smoking at one point in time or another, but the daily smokers had plans for quitting smoking far into the future.

### 3.5. Attempts to Quit

Altogether, 23.5% of the smokers reported that they had made attempts to quit smoking during the past year. Another 47.7% had made attempts to quit more than a year ago or did not remember when an attempt to quit was made. Altogether, 71.2% of the smokers reported that they had made attempts to quit—far more daily smokers (81.7%) than occasional smokers (56.9%). A quarter of smokers (23.5%) had not previously tried to quit smoking.

### 3.6. Index Score

The pairwise correlation among the four indicators was high (Cronbach's alpha 0.815). The fact that the respondents answered consistently (high value of one indicator coincides with high value of other indicators and vice versa) signifies that the indicators measure the same underlying phenomenon (interest in quitting) and that the index thus has good validity. The index range went from 0 (very low interest) to 5 (very high interest), and the average score for the sample was 2.74 (standard deviation 1.363). No significant difference was observed between women and men, between age groups, between regions, or between daily and occasional smokers (see Table B, supplementary materials).

Despite the strong similarity in the average score between the two categories of smokers, differences were observed in the dispersal pattern (variance) of the index. Occasional smokers had a greater accumulation in the extreme categories than the daily smokers who were more evenly distributed (Table 6). Corresponding differences in prevalence were not observed among different age groups or among different regions (not shown in the table). A greater accumulation in the extreme categories was observed among men, compared to women, but the statistical significance of this finding was low (Pearson′s chi − sq. = 11,249, *p* = .024).

In our index, 13.2% of the smokers can be categorized in the group with very high interest in quitting smoking, while a further 19.4% had high interest—all together 32.6%. At the other end, 24.1% of the smokers were classified as having very low interest in quitting smoking and a further 23.7% with low interest—totalling 47.8%. The remaining 19.5% were classified as having an average interest in quitting.

## 4. Discussion

How large a proportion of smokers want to quit and how large a proportion want to continue? As we have demonstrated in this article, the answer depends on which question is asked, how the response categories are grouped together and if we ask smokers with high or low usage intensity. To advance our understanding of smoking cessation behaviour and its association with targeted tobacco policies, we have proposed construction of an index to measure cessation interest in a robust and multidimensional fashion. Using pooled data from the yearly Stoptober campaign, we have also demonstrated the empirical utility of this instrument.

Our findings are fairly congruent with nationally representative data. Just over 40% of the smokers in Statistics Norway's nationally representative surveys answer that they are very interested or fairly interested in quitting smoking [49]. This is consistent with our Table 2. Moreover, the proportion of daily smokers with an intention to quit within six months—approximately 40%— and the proportion who has never attempted to quit — approximately 20% — is also fairly similar in the two surveys. (Tables 4 and 5, respectively).

### 4.1. How Many Smokers Want to Quit?

Previous estimations of the proportion of smokers who want to quit smoking varies according to the indicator used. Norwegian Directorate of Health [50] and Norwegian Cancer Society [51] have previously stated that about 70% of the daily smokers want to quit—an estimate consistent with reports from the US [52]. However, in Norway, such estimate may have emerged from a procedure where only one of the indicators was applied, for instance “to what extent do you want to quit smoking?” (with the mid value (3) on the scale from 1 to 5 being assigned to the group expressing interest in smoking cessation) or measures of the proportion of smokers who has ever attempted to quit. According to our index, however, only 1/3 of the smokers could be categorized in this group, while approximately half were uninterested in quitting smoking. A similar share of smokers uninterested in quitting has been found in Turkey [26].

In addition, we discovered differences between daily and occasional smokers. When daily smokers were asked if they had attempted to quit smoking, 82% answered “yes,” but only 21% reported that this attempt had been made during the past year. When asked if they were considering quitting smoking, 64% of the daily smokers answered “yes,” but only 11% had the intention of quitting in the course of the next month. If asked whether they portray themselves as non-smokers in the future, 48% answered “yes,” but only 24% assumed this would happen within one year. Furthermore, 68% of the daily smokers expressed a desire to quit smoking, but only 16% wanted this to a very large extent.

Among occasional smokers—who comprised about 45% of the smokers—there were generally lower proportions who wanted to quit (59%), who were considering quitting (46%), and who had tried to quit (57%). However, unlike daily smokers, it appears that occasional smokers include a relatively higher proportion who report *immediate intentions* to quit (19%), and have made *recent* attempts to quit (27%). Occasional smokers also more often envisage a smoke-free future (63%) than daily smokers and relatively more (34%) state that this could happen within a year.

### 4.2. Will Interest in Quitting Reflect the Future Rate of Quitting?

For a long time, quitting smoking was considered a sequential process that went through maturing stages based on the transtheoretical model of change for addictive behaviours [32, 53]. However, more recent research indicates that attempts to quit smoking occur from all stages in this model and that presence at one stage predicts which becomes the next only to a limited extent [54–57]. Smokers bounce back and forth, and situational factors trigger spontaneous attempts to quit. In the Stoptober survey, for instance, 30.6% of the daily smokers (*N* = 621) and 50.9% of occasional smokers (*N* = 320) declared that their last attempt to quit smoking was not preplanned (not in the table). Spontaneous attempts from earlier stages are about as (moderately) successful as planned attempts [58–62]. This means that the size of the segment with strong interest in quitting is *not* necessarily a valid indicator for the future rate of quitting.

### 4.3. Targeting Smoking Cessation Polices towards a Heterogeneous Group of Smokers

Consistent with the WHO guidelines [63], Norwegian tobacco policy is aimed at reducing smoking initiation among the young, protecting third parties from passive smoking, and stimulating smoking cessation among established smokers [64]. According to a systematic scale of tobacco control, Norway ranks sixth among 37 European countries, with an especially high score for its tax level and its ban on indoor smoking [65]. Following the requirements of article 14 in the WHO Framework Convention on Tobacco Control, two smoking cessation services are currently in operation in Norway. These are mainly facilitated for smokers who are motivated to quit: first, all GPs are supposed to survey smoking habits and motivation for smoking cessation. Smokers who are registered as motivated are then sent into counselling services in so-called “Healthy Life” centres, with the purpose of providing support for behavioural lifestyle changes. Second, a digital cessation service (slutta.no) is designed to support smoking cessation online.

In addition, a trial scheme is currently in progress, providing free pharmaceutical NRT products to smokers. In September 2020, a three-year government-funded smoking cessation project was launched in a region representative of Norway's demographics (Vestre Viken Health Trust). There, all daily smokers are offered free quit-smoking medicines and cessation guidance at one of the municipal Healthy Life centres in the area [66]. Such measures do not place any restrictions on smokers' options for action. In all, 21 Healthy Life centres participating in the experiment have given a positive reception to the assistance scheme, and the inflow of smokers who are using the offer has been higher than expected. Similar schemes in the United States have been shown to increase the rate of quitting [67–71]. Participation may be driven by an interplay between desires for financial and health gains, but we know little about the smokers' motivations and the overall effects of this intervention in Norway. Unfortunately, it is unlikely to be evaluated, as attempts to establish control groups in the experimental design failed due to the COVID pandemic.

### 4.4. Assessing the Justification for Intensification of Structural Means

The Norwegian tobacco policy vision of a smoke-free society may also influence the norm-climate for smoking and attitudes towards smoking in the population, which in turn might increase the social acceptance for further intensification of structural measures to curb smoking [64, 72]. It is important to the authorities to identify the segments with and without quit-smoking interest, because their respective size matters in terms of justifying structural measures to curb smoking. To achieve the goal of a smoke- or tobacco-free society, the Norwegian government is currently planning to intensify structural measures. Tax hikes, restricting access to outdoor smoking, and reduced availability are among the instruments [64, 73]. The primary focus of this policy is to prevent young people from starting to smoke. In their plans, authorities acknowledge that such measures may appear unwanted, punitive and coercive to established smokers. However, at the same time, authorities also emphasize that a strengthening of the infrastructure for tobacco control can represent a type of “help” to smokers who—due to some sort of decision failure—continue to smoke against their own will.

The help argument is based on the idea that nicotine addiction affects our ability to choose in such a way that the decision whether to light another cigarette is disturbed by signal-controlled ignition reactions (cues and cravings) [74]. Irresistibility and overwhelming desire can cause smokers to act contrary to their own interests and conviction. In these cases, the structural measures provide smokers with golden opportunities to reintroduce self-regulation and to comply with the wishes they themselves had to begin with [72, 75–78].

However, in invoking the help argument, the authorities should demonstrate that the decision failure will result in serious consequences and, moreover, that a desire to quit is widespread among smokers. Failure to pursue a desire to quit smoking can undoubtedly have *serious* consequences for the smoker's future health status. The *diffusion* of interest in quitting will, as we have observed, be dependent on who we ask (daily smokers or occasional smokers) and the questions we choose to elicit information from. From a justification perspective, it will make a big difference if the percentage of smokers who smoke against their own will is 30% or, approximately 70% as claimed by the Norwegian Directorate of Health [50] and Norwegian Cancer Society [51].

In contrast with pedagogical cessation measures, structural measures imply a reduction in the opportunity set for smokers to act [79]. Even though intensification of structural measures is based on a charity principle and a care ideology, it may entail that smokers who wish to continue smoking will face increasing difficulty, loss of welfare, loss of autonomy, and social disqualification [80]. Studies have revealed that proposed restrictions on purchase availability, reduced access to outdoor smoking, and tax hikes on cigarettes have little support among smokers [81–83]. As smokers are overrepresented among those with lower socioeconomic status [84] and those with mental disorders [85], an already vulnerable group will thereby be hit the hardest. Here, the authorities rely on the widely used argument that, for example, tax increases must nevertheless be considered socially progressive—and not regressive—because the reduction in demand will be the greatest in the groups where smoking is most widespread and thus lead to social equalisation in future health status [73].

One should perhaps try to avoid the help argument from becoming a substitute pretext that the authorities use to regulate unwanted behaviour in a situation where their policy lacks the support of the group they are intending to help. On the other hand, the point could also be made that smokers do not realize that these measures will help them, but that they will nevertheless be grateful afterwards. This reasoning is used, for instance, in support of coercive treatment of drug addicts [86]. Structural measures have no doubt been important both for increasing the rate of quitting smoking and for reducing the incidence of smoking among the youth [87, 88]. Consideration for the latter has rightly been especially important. In a situation where the prevalence of daily smoking among young people is about to decline to 1-2%, is it perhaps time for weighing the intended and unintended consequences when discussing the intensified use of structural measures?

### 4.5. Limitations of the Study

The utility of survey data largely depends on the accuracy of respondents' answers. However, current beliefs and retrospective distortions may influence the encoding of desire to quit, prediction of future smoking status, plans to quit, and quit-smoking memories. For example, smokers in England failed to report a substantial proportion of unsuccessful quit attempts [89, 90].

Smoking has gradually become a norm-breaking behaviour, associated with stigma and self-condemnation [9, 11, 91]. Answers to questions about smoking behaviour posed in a survey sent out by the Norwegian Directorate of Health may therefore be influenced by social desirability bias (how one should respond) [92, 93] and injunctive norms (how one thinks others want one to behave) [94, 95] and thus reduce the validity of the measuring instrument. We do not know the extent of any possible overreporting of the interest in quitting smoking. Such biases may be particularly prevalent during national antismoking campaigns such as Stoptober. However, we have only used data from surveys conducted *prior to* the start of the campaign, and these responses should not be affected by this particular intervention.

The answers may also be distorted by dissonance reduction in the sense that the expressed interest in quitting smoking is adapted to fit the behaviour [96]. To the extent that this type of response rationalisation is effective, it will mean that our estimate of the proportion smokers with interest in quitting will appear too low.

The way the index is calibrated will influence the result. In our index, 31.3% of daily smokers and 34.4% of occasional smokers are categorized as having high or very high interest in quitting. Significantly higher proportions—45.5% and 51.0%, respectively—had low or very low interest in quitting. The results might have been different if, for instance, we had access to additional indicators for interest in quitting beyond the ones included in the index, if we had put different (and not equal) weight on the items in the index or if we had assigned ranking scores to the response categories in a different manner.

Our approach is based on equal weighting of the four indicators. Of our indicators, prediction of future smoking status would, according to the literature, have the weakest association with interest in quitting. A downsized weighting of this indicator in the index will result in the proportion of those with interest in quitting being somewhat smaller. A higher weighting of the direct question on interest in quitting in the index would have resulted in a somewhat larger segment of smokers with interest in quitting.

In our analyses, we use parametric statistics (average, see Table A in supplementary materials), which assumes that the index is at ratio level. Our index, however, is composed of variables at the ordinal level. This means that we treat ordinal variables as if they were continuous. This is very common in social science analyses in general and attitude research in particular [97].

The sampling method means that the results in this study are not necessarily representative of the population of smokers in Norway. Also, the data set only to a small extent includes questions about economic vulnerability and material conditions among smokers. Previous studies have found that smoking patterns and cessation behaviour is associated, not only with gender, age, and education (that we have controlled for) but also with sociological factors such as household rules, support of tobacco policy measures, alcohol use, and household welfare indicators [4, 98]. This means that we may have underestimated potential statistical variance in our index by not controlling for all relevant predictors. However, there exist nationwide studies conducted by Statistics Norway that make it possible to study the same phenomenon using representative data. This will be an assignment for future research.

## 5. Conclusion

Previous estimations of the proportion of smokers who want to quit smoking vary according to the indicator used and who responds. Such estimates have been used to justify an intensification of structural measures to increase smoking cessation, by referring to the fact that they may represent a helping hand to smokers who smoke against their own wishes. In our article, we propose an index of interest in quitting based on four indicators: desire to quit, prediction of future smoking status, plans to quit, and actual attempts to quit. Using this index, we found that one in three smokers reported interest in quitting smoking, while almost 50% were uninterested. For the latter group, intensification of structural measures to curb smoking is likely to be perceived as undesirable. In formulating Norwegian tobacco policy, their views have been given little weight.

A job for future researchers will be to create an index based on indicators of interest in quitting in the nationally representative surveys that Statistics Norway carries out on behalf of the Norwegian Institute of Public Health. Here, it will be possible to analyze the interest in quitting in the light of background variables that are not found in the Stoptober survey, for example, risk perceptions and smoking history. For the authorities, it will be a challenge to create a tobacco policy which also takes into consideration smokers who want to continue to use nicotine. Intensification of structural measures might appear easier to justify if the authorities at the same time facilitate the transition to harm-reducing alternatives for recreational use of nicotine.

## Figures and Tables

**Table 1:** Composition of respondent group based in demographics and smoking status (*N* = 1377).

	Number	Per cent
Gender		
Women	736	53.4
Men	641	46.6
Age group		
18-38 years of age	472	34.2
39-55 years of age	462	33.6
56 years +	443	32.2
Region		
Oslo/capital area	347	25.2
Eastern Norway	392	28.5
Southern and Western Norway	380	27.6
Northern Norway	258	18.7
Smoking status		
Daily	760	55.2
Occasionally	617	44.8

**Table 2:** Extent of desire to quit (“To what extent do you want to quit smoking?”) by smoking status.

Extent of desire to quit	Smoking status			Index score
	Daily (%)	Occasional (%)	All smokers (%)	
To a very great extent	15.9	16.2	16.0	5
To a great extent	24.1	19.6	22.2	4
To some extent	27.6	23.2	25.7	3
To a small extent	13.7	15.5	14.5	2
To a very small extent	16.3	20.5	18.1	0
Do not know	2.4	5.0	3.5	1

Total (*N* = )	100 (760)	100 (561)	100 (1321)	

Pearson's chi-squared test 15,660; significance 0.008.

**Table 3:** Prediction of future smoking status (“Do you see yourself as a nonsmoker in the future?”) by smoking status.

Prediction of future smoking status	Smoking status			Index score
	Daily (%)	Occasional (%)	All smokers (%)	
Yes…	(48.4)	(62.6)	(54.3)	—
Within 12 months	23.7	33.9	28.0	5
Within 1-2 years	10.0	6.8	8.6	4
Within 3-4 years	5.1	3.9	4.6	3
In 5 years or later	2.5	3.7	3.0	2
But do not know when	7.1	14.3	10.1	1
No	17.2	17.1	17.2	0
Do not know	34.3	20.3	28.4	0.5

Total (*N* = )	99.9 (760)	100 (561)	99.9 (1321)	

Pearson's chi-squared test 57,159; significance 0.000.

**Table 4:** Plans to quit smoking (“Do you have plans to quit smoking?”) by smoking status.

Plans to quit	Smoking status			Index score
	Daily (%)	Occasional (%)	All smokers (%)	
Considering quitting smoking…	(63.9)	(46.3)	(56.4)	—
In the course of next month	10.9	18.7	14.2	5
In 1-3 months	16.2	13.4	15.0	4
In 4-6 months	13.4	6.4	10.4	3
But not in the course of the next 6 months	23.4	7.8	16.8	2
Are not considering quitting smoking	19.7	32.8	25.3	0
Do not know, do not wish to answer	16.3	20.9	18.2	1

Total (*N*=)	99.9 (760)	100 (561)	99.9 (1321)	

Pearson's chi-squared test 102,675; significance 0.000.

**Table 5:** Last attempt to quit smoking (“Have you tried to quit smoking?”) by smoking status.

Attempt to quit	Smoking status			Index score
	Daily (%)	Occasional (%)	All smokers (%)	
Yes….	(81.7)	(56.9)	(71.2)	—
Last month	3.4	6.2	4.6	5
1-3 months ago	4.2	8.2	5.9	4.5
4-6 months ago	5.3	6.4	5.8	4
7-12 months ago	8.3	5.7	7.2	3.5
1-2 years ago	13.4	9.4	11.7	3
More than 2 years ago	46.3	17.8	34.2	2
Yes, but do not remember when	0.8	3.2	1.8	1
No	16.2	33.5	23.5	0
Do not remember	2.1	9.4	5.2	0.5

Total (*N*=)	100 (760)	99.8 (561)	99.9 (1321)	

Pearson's chi-squared test 183,771; significance 0.000.

**Table 6:** Smokers' cessation interest index scores based on the four quitting indicators (percent).

Interest in quitting	Smoking status		
	Daily	Occasional	All smokers
Very high (4.1-5)	10.4	17.1	13.2
High (3.1-4)	20.9	17.3	19.4
Medium (2.1-3)	23.2	14.6	19.5
Low (1.1-2)	25.0	21.9	23.7
Very low (0-1)	20.5	29.1	24.1

Total (*N* = )	100 (760)	100 (561)	99.9 (1321)

Pearson's chi-squared test 36,255; significance 0.000.

## Data Availability

Data used in this article are the property of the Norwegian Directorate of Health and are not publicly available.

## Supplementary Materials

Supplementary Materials Two supplementary tables: Table A (Smoking status by year) and Table B (average score of index of interest in quitting smoking).
