# Supplementary material for: Indexing Quit-Smoking Interest among Norwegian Smokers 2019-2021
Source: J Smok Cessat. 2023 Feb 13;2023:9536270. doi: 10.1155/2023/9536270 (PMC9940954; doi:10.1155/2023/9536270)
Supplement: Supplementary Materials — Two supplementary tables: Table A (Smoking status by year) and Table B (average score of index of interest in quitting smoking). [file 9536270.f1.docx]

**Supplementary file: Appendix**

*Table A. Smoking status by year. Gross respondence.*

|  | **2019** | **2020** | **2021** |
| --- | --- | --- | --- |
| Do you smoke (cigarettes, RYO, pipe, cigars/cigarillos)? |  |  |  |
| Yes, daily | 36.8 | 40.4 | 41.9 |
| Yes, occasionally | 34.4 | 31.9 | 31.7 |
| No, I have quit* | 28.8 | 27.7 | 26.4 |
| (N=)  (N current smokers=)** | (500)  (356) | (700)  (506) | (700)  (515) |

**=excluded from the study, **=included in the study.*

*Table B. Average score of index of interest in quitting smoking.*

|  | Average | Standard  deviation | N |
| --- | --- | --- | --- |
| All | 2.74 | 1.363 | 1321 |
| Gender |  |  |  |
| Women | 2.75 | 1.315 | 712 |
| Men | 2.72 | 1.419 | 609 |
| Smoking status |  |  |  |
| Daily | 2.76 | 1.280 | 760 |
| Occasional | 2.71 | 1.470 | 561 |
| Region |  |  |  |
| Oslo/Capital area | 2.63 | 1.411 | 334 |
| Eastern Norway | 2.79 | 1.360 | 381 |
| Southern and Western Norway | 2.80 | 1.355 | 358 |
| Northern Norway | 2.73 | 1.312 | 248 |
| Age group |  |  |  |
| 18-38 years of age  39-55 years of age  56 years + | 2.69  2.79  2.73 | 1.373  1.337  1.381 | 432  449  440 |
